# Supplementary material for: Field-Evolved ΔG210-ppo2 from Palmer Amaranth Confers Pre-emergence Tolerance to PPO-Inhibitors in Rice and Arabidopsis
Source: Genes (Basel). 2022 Jun 10;13(6):1044. doi: 10.3390/genes13061044 (PMC9222656; doi:10.3390/genes13061044)
Supplement: Supplementary file 1 [file genes-13-01044-s001.zip › genes-1652798-supplementary.pdf]

Field-evolved *ΔG210-ppo2* from Palmer Amaranth confers preemergence tolerance to PPO-inhibitors in rice and Arabidopsis

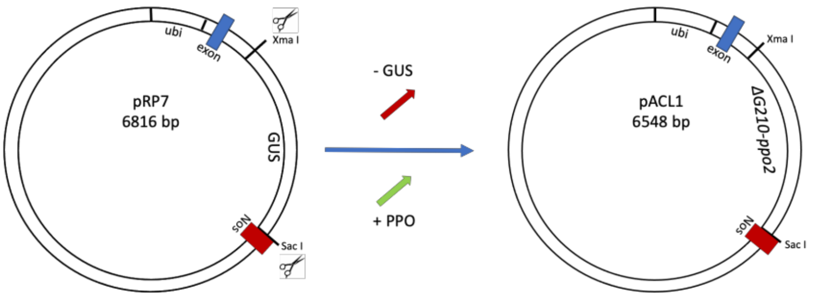

Figure S1: Schematic diagram showing construction of pACL1 used for rice transformation

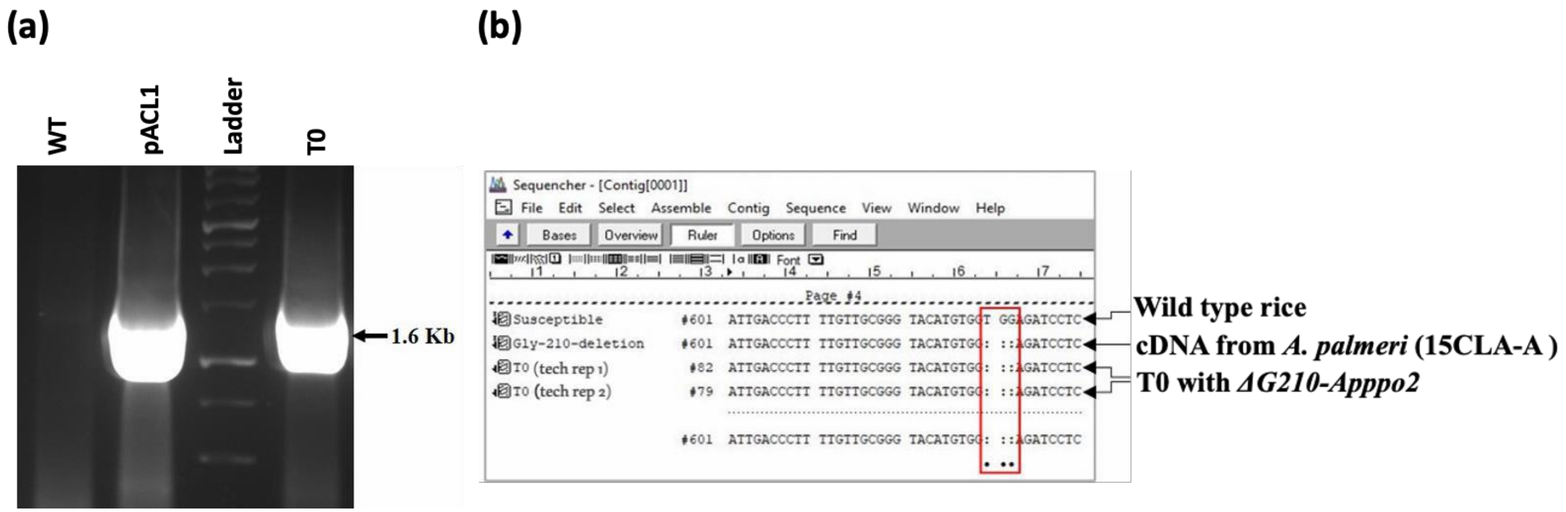

Figure S2: Confirmation of presence of transgene carrying *ΔG210-Apppo2* **(a)** Detection of *ΔG210-Apppo2* transgene in rice genomic DNA by PCR amplification. The PCR products (seen as bands) were generated using Palmer amaranth *ppo2* primer pair (KpnF and SphR) flanking a 1.6kb region encoding *A. palmeri ppo2*. **(b)** Nucleotide sequence alignment of protoporphyrinogen IX oxidase (*PPO2*) in wild type (Susceptible), resistant (Gly- 210-deletion), and transformed survivor (T_0 fragment 1 and 2). Transgenic plant DNA fragments harbored *ΔG210*. *ΔG210* position is enclosed in the red box.

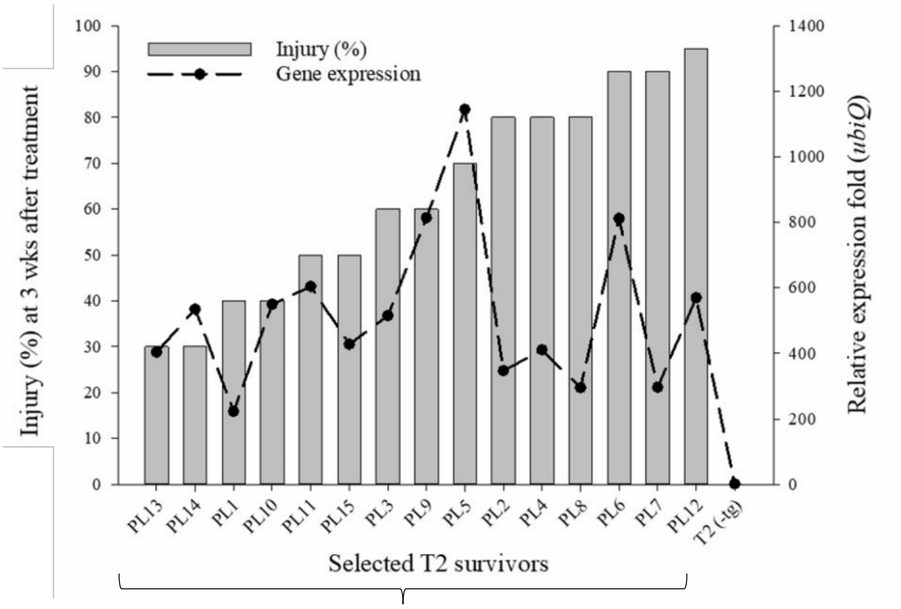

Figure S3: Visible injury (%) and transgene expression of T2 plants surviving 390 g ai ha^{-1} soil-applied fomesafen. Transgene expression is calculated relative to rice ubiquitin. Nontreated T_2(-tg) is used as control for transgene expression.

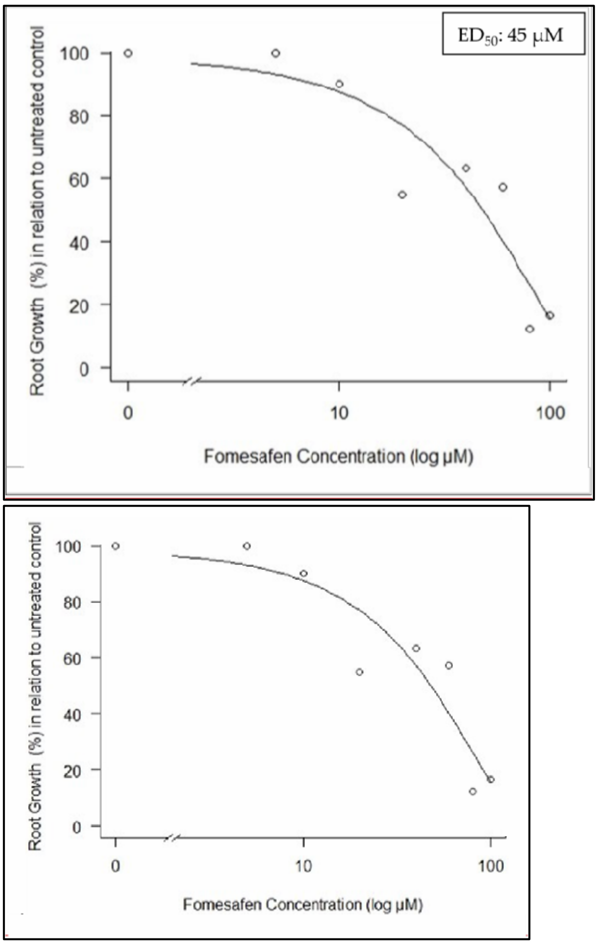

Figure S4: Transformed rice (T_2) seedling root growth assessment (%) relative to the nontreated check as affected by increasing concentration of fomesafen in agar medium. A non-linear, three-parameter log-logistic regression function $Y=d/1+\exp\{[\log(x) - \log(ED50)]\}$ was fitted to the data.

Table S1: Primer sequences used in the transgene expression analysis of rice transformants by qPCR.

Targeted Gene	Primer Sequence	Primer Name
A. palmeri ppo2 containing *ΔG210* mutation	5'-AGGAAAAGGGTGGAGGAGAA-3'	q*PPO2*F4
	5'-GGACAGCACCTCACACTGG-3'	q*PPO2*R4
O. sativa native *PPO2*	5'-TGGTAACGTGAAGCTTGGTACA-3'	*OsPPO2*F2
	5'-CAGAAATTGACCAACCACCA-3'	*OsPPO2*R2
Ubiquitin	5'-CGCAAGTACAACCAGGACAA-3'	*ubiQ*F
	5'-GCTGTGACCACACTTCTTCTT-3'	*ubiQ*R

Table S2: Hierarchal clustering of T_2 rice survivors based on injury levels (%) at 3 weeks after preemergence treatment with fomesafen at 390 g ha^{-1}

Cluster	No. of individuals	Injury (%)		
		Mean	Min	Max
1[a]	6	40	30	50
2[b]	6	65	60	70
3[c]	12	88	80	95

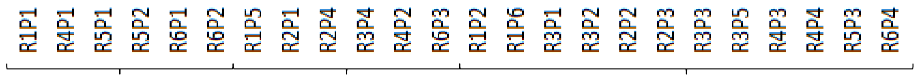

Cluster 1 Cluster 2 Cluster 3

[a] Cluster 1 = highly tolerant to fomesafen with injury <50% (6 individuals).
[b] Cluster 2= moderately tolerant to fomesafen with injury ranging from 60 to 70%.
[c] Cluster 3= slightly tolerant to fomesafen with injury ranging from 80 to 95%.

Table S3: Dose response of transgenic *Arabidopsis* line to soil-applied fomesafen

**Dose (g ha^{-1})**	***Arabidopsis* (*ΔG210-Apppo2*)**		**Wild type (Control)**	
**Fomesafen**	**% Emergence**	**SD[a]**	**% Emergence**	**SD**
0.08	51.5	2.5	49	1.5
0.25	55	3	39	2
0.74	42	3.5	11.5	3
2.22	39	3.5	0	6
6.67	39	0.5	0	9
20	32.5	0.5	0	2.5
60	16	0	0	6
120	2.5	0	0	1.5

[a]SD = standard deviation

Table S4: Dose response of transgenic *Arabidopsis* line to soil-applied saflufenacil.

**Dose (g ha^{-1})**	***Arabidopsis* (*ΔG210-Apppo2*)**		**Wild type (Control)**	
**Saflufenacil**	**% Emergence**	**SD[a]**	**% Emergence**	**SD**
0.25	56.5	4	49	1.5
0.74	46	3	38	1
2.22	41.5	1.5	16.5	1.5
6.67	38	0	0	3
20	35	0	0	6
60	28	0	0	3
120	13	0	0	1

[a]SD = Standard deviation
